# Clinical Features and Outcomes of Repeated Endoscopic Therapy for Esophagogastric Variceal Hemorrhage in Cirrhotic Patients: Ten-Year Real-World Analysis

**DOI:** 10.1155/2020/5747563

**Published:** 2020-05-15

**Authors:** Jia-li Ma, Ling-ling He, Ping Li, Yu Jiang, Ju-long Hu, Yu-ling Zhou, Xiu-xia Liang, Hong-shan Wei

**Affiliations:** Department of Gastroenterology, Beijing Ditan Hospital, Capital Medical University, Beijing, China

## Abstract

**Objective:**

This study is aimed at evaluating the survival of cirrhotic patients with different etiologies after endoscopic therapy for acute variceal bleeding and the effect of repeated endotherapy on patients' prognosis.

**Methods:**

We retrospectively evaluated the clinical features and outcomes between cirrhotic patients with chronic HBV or HCV infections and other etiologies. The 3-year and 5-year survival rates and rehemorrhage rate in one year between the viral and nonviral cirrhosis patients were compared by Kaplan-Meier curves and log-rank test. Cox analysis was used to identify the impact factors that affect the long-term survival of patients with cirrhosis and variceal bleeding after endotherapy.

**Results:**

Out of 2665 patients with liver cirrhosis and variceal hemorrhage selected from our medical center between September 2008 and December 2017, a total of 1342 patients were included for analysis. The median follow-up duration was 32.9 months (range 0.16-111.4 months), the 3- and 5-year cumulative survival rates were 75.3% and 52.8%, respectively. The median survival time was significantly longer in viral cirrhosis patients (47.1 months [95% CI: 24.9-69.1]) compared with nonviral cirrhosis patients (37.0 months [95% CI: 25.0-56.0], *p* = 0.001). The 3-year and 5-year survival rates of the viral group were higher than the nonviral group. The rehemorrhage rate at one year was higher in nonviral patients than in viral patients (*p* < 0.001).

**Conclusion:**

Repeated endotherapy combined with effective antiviral therapy is helpful for long-term survival of cirrhotic population with variceal hemorrhage and HBV or HCV infection.

## 1. Introduction

Acute variceal bleeding (AVB) is a fatal complication in patients with liver cirrhosis, which is associated with increased mortality of about 20% at six weeks, despite recent progress in management. Nearly 50% of patients with newly diagnosed liver cirrhosis have accompanying varices, and new varices develop every year or preexisting varices worsen in 7-8% of patients, and the first bleeding occurs in 12% of patients each year [1, 2]. Most cirrhotic patients cannot receive liver transplantation after variceal hemorrhage and other cirrhotic complications in China, since few donated livers are used and it is too expensive for most patients to receive transplantation. Consequently, endoscopic therapy is one of most common techniques to control esophageal variceal bleeding in such patients. However, the clinical outcomes of cirrhotic patients with different etiologies after repeated endotherapy for AVB remain unknown.

The past 2–3 decades have seen significant advances in the management of portal hypertension and AVB, and this has led to marked improvement in survival [3–5]. The hemostatic rate achieved by emergency EIS ranged between 60 and 100% [6]. EVL has been extensively evaluated in the treatment of variceal bleeding and in the prophylaxis of first bleeding [7, 8]. Cyanoacrylate injection is globally accepted as the primary intervention for gastric variceal bleeding. It was reported that 92% of patients admitted for acute gastric variceal bleeding achieved successful hemostasis after the injection of cyanoacrylate [9]. Combined treatment with vasoactive drugs, prophylactic antibiotics, and endoscopic techniques is the recommended standard of care for patients with AVB [10]. Endoscopic therapy has been considered the mainstay treatment for AVB. EVL combined with a vasoactive drug is considered the standard care for AVB, which is recommended by BAVENO VI [11]. However, the long-term effect of ligation plus cyanoacrylate injection with or without sclerotherapy of variceal bleeding in cirrhotic patients with concomitant esophageal and gastric varices remains unclear.

In the present study, we retrospectively analyzed 1342 liver cirrhosis patients with variceal bleeding after endotherapy in our hospital during the past decade. The aim of this study was to analyze patients with cirrhosis receiving standard therapies for the different etiologies and complications in a real-life setting. Survival and rebleeding analysis was used to evaluate the prognostic impact of viral liver cirrhosis and nonviral cirrhosis. Factors that affect the clinical outcomes were analyzed in patients with liver cirrhosis and variceal bleeding after endoscopic therapy.

## 2. Patients and Methods

### 2.1. Subjects

This retrospective single-center study was completed at the Beijing Ditan Hospital of Capital Medical University. The clinical data was collected between September 2008 and December 2017. Eventually, 1342 liver cirrhosis patients with variceal bleeding who were treated with endotherapy were included in the analysis. The exclusion criteria were as follows: (i) patients with liver carcinoma and other serious concurrent illnesses; (ii) patients who received TIPS, splenic embolization, pericardial devascularization with splenectomy, or other interventions for varices; and (iii) patients who were lost to follow-up.

The study protocol was approved by the Ethics Committee of Beijing Ditan Hospital, Capital Medical University [JDLKZ (2018-021-01)]. The study has been registered in the Chinese Clinical Trial Registry (ChiCTR1800019265).

### 2.2. Data Collection

Baseline characteristics included clinical characteristics and laboratory values, such as white blood cell (WBC), red blood cell (RBC), platelet (PLT), lymphocyte (LYM), neutrophilic granulocyte percentage (NEU%), hemoglobin (HGB), alanine aminotransferase (ALT), aspartate transferase (AST), cholinesterase (CHE), gamma-glutamyl transpeptidase (GGT), total bilirubin (TBIL), albumin (ALB), creatinine (Cr), prothrombin time (PT), and alpha-fetoprotein (AFP). Transabdominal ultrasonography values such as portal vein diameter and spleen thickness were also collected. Child-Pugh score and MELD score were recorded. All of the baseline data were collected at the first endotherapy for variceal bleeding. The etiology of the patients was also recorded.

### 2.3. Treatments and Endoscopic Therapy

All patients with advanced cirrhosis and AVB were started on vasoactive drugs including vasopressin, somatostatin, and their analogs as the initial treatment before endotherapy. Antibiotics were also administered as soon as the patients arrived at the hospital. Endotherapy was performed when the patients achieved hemodynamic stability, 12-24 hours after hospital admission.

The endotherapy was performed using the following methods: (i) endoscopic variceal ligation (EVL) was used for patients with the first esophageal variceal bleeding; (ii) cyanoacrylate injection was used for patients with gastric variceal hemorrhage; (iii) the “sandwich therapy” was performed in patients with esophageal gastric variceal hemorrhage. Patients were injected with 2 mL polidocanol, 0.5 mL n-butyl-2 cyanoacrylate, and 2 mL polidocanol (repeated injection if necessary). Most patients with GOV1 and GOV2 varices received repeated endotherapy every 3-6 months during follow-up. Endoscopic injection sclerotherapy (EIS) was also performed for some patients with esophageal variceal rebleeding based on the clinical guidelines [12, 13] and the specialists' preference and experience for choosing the best endotherapy. Nonselective beta blockers (NSBBs) were also administered for the secondary prevention of rebleeding after achieving hemodynamic stability.

### 2.4. Follow-Up

The follow-up ended in September 2017. The primary outcome was 3- and 5-year survival. The secondary outcome was rebleeding rate within one year. The median follow-up duration was 65 months. The longest follow-up duration was 126 months.

### 2.5. Statistical Analysis

The dataset was divided into different groups for analysis. The clinical characteristics of viral and nonviral cirrhosis patients were analyzed. Quantitative data was detected for normal distribution. Student's *t*-test was used if the data accorded with normal distribution, and the Mann-Whitney *U* test was used if the data did not accord with normal distribution. Mean ± standard deviation and interquartile range were used as appropriate. The chi-square test was used for analyzing qualitative data, and the data were expressed in percentage.

To examine the survival differences among the groups, the Kaplan-Meier method was used to generate the survival curves. The log-rank method was used to compare the differences between the groups. Univariate and multivariate Cox analyses were conducted to identify the impact factors that influenced the long-term survival. Two-sided *p* values < 0.05 were considered statistically significant. Statistical analyses were performed using SPSS 19.0 (IBM Corp., Armonk, NY, United States) and GraphPad Prism 7.0 (GraphPad Prism Software Inc., San Diego, CA, USA).

## 3. Results

### 3.1. Clinical Characteristics

A total of 2665 patients with liver cirrhosis and variceal bleeding after endotherapy were assessed in the study. After excluding 363 patients lost to follow-up; 376 patients with preexisting HCC and other serious concurrent illnesses; and 584 patients who received TIPS, splenic embolism, splenectomy, and liver transplantation, 281/584 patients received these interventions after repeated endotherapy, and these patients were further interviewed, of which 78 patients received the intervention accompanied with indications for other treatments, 203 patients were for other reasons, such as being combined with cholecystolithiasis requiring cholecystectomy; being combined with suspected malignant nodules; and personal preference or experience of the physician. A total of 1342 patients were included in the study. Of these, 930 patients had viral cirrhosis (including 828 CHB patients and 102 CHC patients) and 412 patients had nonviral cirrhosis. Of the 412 patients with nonviral cirrhosis, 95 patients had cryptogenic cirrhosis, 211 patients had alcoholic cirrhosis, 94 patients had autoimmune cirrhosis, and 12 patients had other diseases (two with Wilson's disease, eight with drug-induced liver cirrhosis, one with Sjogren's syndrome-related cirrhosis, and one with hematologic disease-related cirrhosis).

**Figure 1:**
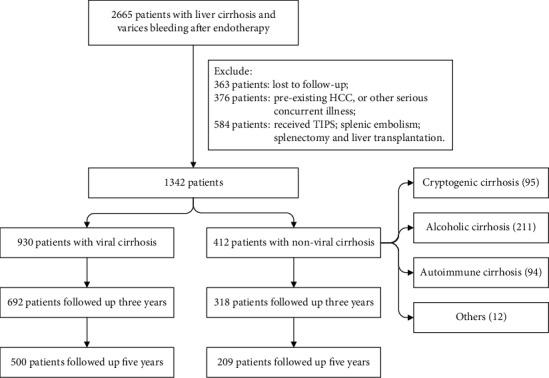
Flowchart of patient selection process.

Among the 1342 patients included in the study, 68.5% (919 of 1342) were male, and the average age was 52.2 years (range 18-83 years). The median follow-up duration was 65 months (range 0.16-126.40 months), and the 3- and 5-year cumulative survival rates were 75.3% and 52.8%, respectively. A total of 1010 patients were followed up for three years, of which 692 had viral cirrhosis and 318 had nonviral cirrhosis, while nine patients died of noncirrhosis-related causes. A total of 709 patients were followed up for five years, of which 500 had viral cirrhosis and 209 had nonviral cirrhosis. The flowchart of the patient selection process is shown in Figure 1. The baseline characteristics of patients who were followed up for three years are shown in Table 1.

### 3.2. Comparison of Survival between Viral and Nonviral Populations

**Figure 2:**
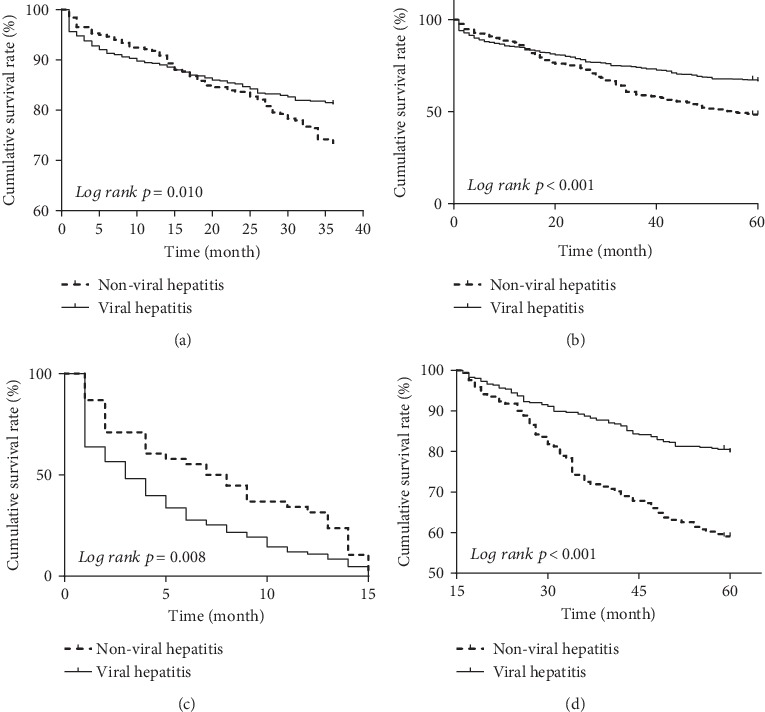
The cumulative survival rates in viral and nonviral hepatitis. (a) The 3-year cumulative survival rates in viral and nonviral hepatitis. (b) The 5-year cumulative survival rates in viral and nonviral hepatitis. (c) The cumulative survival rate for the first 15 months after endotherapy in viral and nonviral hepatitis. (d) The cumulative survival rate from 15 to 60 months after endotherapy in viral and nonviral hepatitis.

To evaluate the long-term outcome of endotherapy, the survival rates between the viral and nonviral groups were analyzed. The result showed that the median survival time was significantly longer in viral cirrhosis patients (47.1 months [95% CI: 24.9-69.1]) compared with nonviral cirrhosis patients (37.0 months [95% CI: 25.0-56.0]) (*p* = 0.001). The 3-year survival rate of the viral group was 81.2%, which was higher than that of the nonviral group (73.3%) (*p* = 0.01) (Figure 2(a)). The 5-year survival rate of the viral group was 66.6%, which was significantly higher than that of the nonviral group (48.3%) (*p* < 0.001) (Figure 2(b)). The viral cirrhosis patients had longer survival time than the nonviral cirrhosis patients. However, there was an intersection in the survival curves of both groups at 15 months after endotherapy. So we further compared the survival rate before and after 15 months in the viral and nonviral groups. The result showed that the survival rate was significantly higher in the nonviral group than in the viral group within 15 months after patients received endotherapy (*p* = 0.008) (Figure 2(c)). In contrast, the survival rate was significantly higher in the viral group than in the nonviral group after 15 months after endotherapy (*p* < 0.001) (Figure 2(d)).

Since different etiological factors may influence the prognosis of cirrhosis, we further assessed the survival rates of subgroups of nonviral cirrhosis patients.

**Figure 3:**
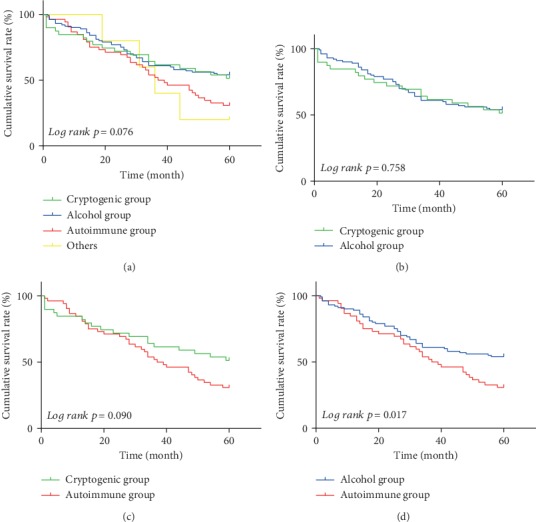
The cumulative survival rates in subgroups of nonviral cirrhosis patients. (a) The cumulative survival rates in all subgroups of nonviral cirrhosis patients. (b) The cumulative survival rates in the cryptogenic group and the alcohol group. (c) The cumulative survival rates in the cryptogenic group and the autoimmune group. (d) The cumulative survival rates in the alcohol group and the autoimmune group.

In the cryptogenic group, the 1-, 3-, and 5-year cumulative survival rates were 93.7%, 73.7%, and 51.3%, respectively. In the alcohol group, the 1-, 3-, and 5-year cumulative survival rates were 94.8%, 75.5%, and 54.0%, respectively. In the autoimmune group, the 1-, 3-, and 5-year cumulative survival rates were 91.5%, 65.3%, and 30.8%, respectively. There were no significant differences in the 1-year, 3-year, and 5-year, survival rates among the four subgroups (*p* = 0.076) (Figure 3(a)). The survival rates of the subgroups were further compared in pairs (Figures 3(b)–3(d)); the 5-year survival rate differed between the alcohol group and the autoimmune group (*p* = 0.017) (Figure 3(d)).

### 3.3. Comparison of Rebleeding between Viral and Nonviral Populations

**Figure 4:**
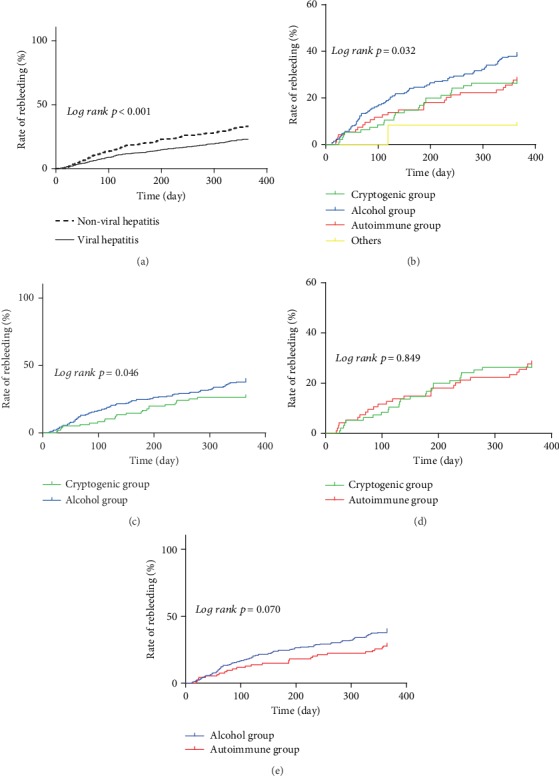
The cumulative rebleeding rates in various groups. (a) The cumulative rebleeding rates in the viral group and the nonviral group. (b) The cumulative rebleeding rates in subgroups of non-viral cirrhosis patients. (c) The cumulative rebleeding rates in the cryptogenic group and the alcohol group. (d) The cumulative rebleeding rates in the cryptogenic group and the autoimmune group. (e) The cumulative rebleeding rates in the alcohol group and the autoimmune group.

The rebleeding rate at one year was compared between the viral and nonviral groups. The viral population had a lower rebleeding rate than the nonviral population (23.0% versus 33.0%, *p* < 0.001) (Figure 4(a)).

The rebleeding rate at one year was also different in the subgroups of the nonviral population (*p* = 0.032) (Figure 4(b)). When we further compared it in pairs, the rebleeding rate was significantly higher in the alcohol group than in the cryptogenic group (*p* = 0.046) (Figure 4(c)).

### 3.4. Predictors of Long-Term Survival

Among the 1342 liver cirrhosis patients with AVB after endotherapy, 709 patients were followed up for five years, of whom 500 were viral cirrhosis patients, while 209 were nonviral cirrhosis patients. Factors associated with 5-year mortality were analyzed by univariate and multivariate Cox regression analyses. Univariate analysis showed that age, gender, WBC, NEU%, LYM%, HGB, GGT, TBIL, ALB, Cr, INR, MELD score, and Child-Pugh score significantly impacted the incidence of death in five years. Multivariate analysis showed that age, LYM%, HB, GGT, DBIL, ALB, and MELD score were independent impact factors for the high risk of mortality (Table 2).

## 4. Discussion

Endoscopic therapy has been used to treat variceal hemorrhage for nearly half century. However, the long-tern survival of cirrhotic patients with repeated variceal bleeding remains unknown. Liver transplantation is unavailable for the majority of cirrhotic patients in China. When these patients suffer from AVB, endotherapy is their major treatment option. It is necessary to evaluate the survival after repeated endotherapy in liver cirrhosis patients with AVB. In this study, survival analysis was performed to evaluate the prognosis of viral and nonviral liver cirrhosis patients after endotherapy.

Westaby et al. conducted a prospective, randomized, controlled trial of injection sclerotherapy in the long-term management of variceal bleeding in 1977. They found a significant improvement in survival in the sclerotherapy-treated patients [14]. After variceal obliteration was achieved, episodes of rebleeding were reduced by almost 10-fold, which showed that patients benefit from endotherapy, since variceal bleeding was the leading cause of death in such patients.

The median survival after decompensation was 2-4 years, which was demonstrated by the first appearance of variceal bleeding, ascites, encephalopathy, and jaundice [15]. A cohort study of 174 patients hospitalized for GOV bleeding, with an overall mean follow-up of 22 months, showed that the cumulative rebleeding rate at one year was 30% and the cumulative survival rates at 1 year and 5 years were 73.4% and 37%, respectively [16]. Another study enrolled 39 patients with Child C who had undergone endoscopic treatment and showed that the 3-year survival was 28.2% [17]. Yet another study enrolled 49 patients, with a mean follow-up duration of 30 months and showed that the rebleeding rate at one year after endotherapy was 45% [18]. In this study, which had longer follow-up duration and larger sample size, the 3- and 5-year cumulative survival rates were 75.3% and 52.8%, respectively, while the rebleeding rate at one year was 33% in nonviral cirrhosis patients and 23% in viral cirrhosis patients. The prognosis seemed better than previously reported studies. For patients with repeated variceal hemorrhage, endotherapy may be helpful for long-term survival. Meanwhile, the exclusion of preexisting HCC may have a slight effect on the results.

The most important finding of this study was that the viral cirrhosis population had a higher survival rate than the nonviral cirrhosis population. In the 15 months after endotherapy, the survival rate was higher in the nonviral cirrhosis population, but after 15 months, it was higher in the viral cirrhosis population. The reason might be that the long-term use of antiviral treatment could improve the patients' survival. The rebleeding rate was much lower in the viral cirrhosis patients than in the nonviral cirrhosis patients.

In China, most of the viral cirrhosis patients suffer from CHB, and the approval of oral antiviral agents has greatly improved the prognosis [19, 20]. In CHB patients, it was reported that hepatic decompensation was associated with high mortality and the 5-year survival rate was 14-35% [21], and the overall survival was 80% at two years after the onset of decompensation [22]. In 2015, Jang et al. reported that patients with decompensated hepatitis B virus-related cirrhosis who took early antiviral treatment had a 5-year survival probability of 59.7% [23], which was lower than 67.1% reported in the present study. This may be owing to improved medical management of cirrhosis and the repeated endotherapy procedures. With standardized antiviral therapy and repeated endotherapy, fewer patients died of AVB, thus improving the survival rate.

We further analyzed the survival difference between each subgroup in the nonviral cirrhosis population. The long-term survival rate in the autoimmune cirrhosis patients was much lower, while the survival rates of cryptogenic cirrhosis patients and alcoholic cirrhosis patients were nearly identical. The rebleeding rate was significantly higher in the alcoholic cirrhosis patients than in the cryptogenic cirrhosis patients and autoimmune cirrhosis patients.

Cryptogenic cirrhosis is a diagnosis of exclusion when there is no other known identifiable etiology [24]. It is suggested that many cryptogenic cirrhosis patients may have evolved from NASH [25]. In this study, there was a high proportion of patients with cryptogenic cirrhosis (94 patients). The reason for this is that our medical center is a national referral center for liver diseases, so the number of unexplained cirrhosis patients referred to our center is far more than those of other hospitals. We interviewed and studied these patients and found that a large proportion of these cryptogenic cirrhosis patients had type 2 diabetes mellitus (66/95) and present or previous obesity (41/95). Hence, we hypothesized that the majority of cryptogenic cirrhosis patients in this study were actually “burnt-out NASH”.

In the present study, most of the autoimmune liver cirrhosis patients were primary biliary cirrhosis (58.5%). A unique feature of primary biliary cirrhosis is the development of varices before the onset of cirrhosis. Esophageal varices develop in about a third of patients with stages 3-4 over a median period of 5-6 years, and roughly half of these patients will have a bleeding event. The development of esophageal varices has a huge impact on survival. The 3-year survival after initial variceal bleeding is about 50% [26, 27]. Esophageal varices were a predictor of higher mortality risk, which may be the reason for the low survival rate of the autoimmune subgroup.

Alcoholic liver cirrhosis is a “malignant” disease with an unfavorable prognosis involving a mortality of 49% and 90% after 1 and 15 years of follow-up, respectively. Almost 60% of patients died as a result of bleeding, hepatic coma, or a combination of these two complications. The 5-year survival of alcoholic liver cirrhosis with variceal bleeding was 27% [28, 29]. Another population-based cohort study of 11873 persons with diagnosis of alcoholic liver disease found that the absolute 5-year survival rate in alcoholic liver cirrhosis was 0.29 (95% CI: 0.28-0.30). The majority of alcoholic liver disease patients (65%) died from alcohol-related cause, i.e., alcoholic liver disease and its direct complications [30]. The high proportion of persistent drinkers among patients with variceal bleeding contributed to the high mortality. Abstinence is the simplest way to control the etiology. Among the 211 alcoholic cirrhotic patients, 148 were nondrinkers while 63 were drinkers, the median survival time of the abstinence group was 30.6 months (95% CI: 22.6-38.7), and the median survival time of the drinking group was 33.2 months (95% CI: 23.2-43.2), with no significant differences between the two groups (*p* = 0.675). Besides, logistic regression analysis showed that neutrophils is the only risk factor for variceal rebleeding (univariate analysis: *p* = 0.022, OR = 1.128, 95% CI: 1.017-1.25; multivariate analysis: *p* = 0.026, OR = 1.152, 95% CI: 1.017-1.300). No dose-response relationship was found between alcohol consumption and liver injury, so alcohol consumption may have no effect on variceal rebleeding [31].

As in previous studies, indicators of the severity of hemorrhage (hemoglobin) and severity of liver dysfunction (MELD score, albumin, and bilirubin) were prognostic factors for cirrhotic patients with variceal bleeding. These factors were predictors for short-term outcomes (such as 5-day treatment failure and 6-week mortality), as well as long-term outcome (5-year mortality) [32]. The predictive role of GGT for 5-year mortality may suggest liver damage. GGT is routinely used in clinical practice as an indicator of liver injury, and plays an important role in protecting cells against oxidants produced during normal metabolism [33]. GGT is also associated with the risk of all-cause mortality [34]. NEU% but not LYM% was included in the factors associated with 5-year mortality in the multivariate analysis, perhaps because LYM% mainly indicates hypersplenism and indirectly responds to portal hypertension. Indicators of 5-year mortality were related to liver disease per se rather than the severity of bleeding in general.

This study had several limitations. First, due to its retrospective study design, there may have been missing data due to recall bias among patients who were contacted by telephone. Second, endotherapy was strictly in accordance with the guidelines, no data suggested that combined endotherapy has any effect on rebleeding rate, so the GOV type and treatment option were not considered in this study. Third, the study was performed in a single center, and the choice of endoscopic treatment had subjective bias. Hence, further verification in other centers is needed. Fourth, the aim of this retrospective study was to evaluate the survival of patients with repeated endotherapy. Patients with other interventions for varices were excluded, 363 patients were lost to follow-up, and there were slight differences in MELD score between the viral and nonviral groups (4 vs. 6). Hence, selection bias may exist.

## 5. Conclusion

Repeated endotherapy combined with effective antiviral therapy is helpful for long-term survival of cirrhotic population with variceal hemorrhage and HBV or HCV infection.

## Figures and Tables

**Table 1:** Baseline characteristics of patients followed up for 3 years.

Variable	Nonviral (*n* = 318)	Viral (*n* = 692)	*p*
Age (year)	55.3 ± 12.5	50.4 ± 10.6	<0.001
Male sex	208 (65.4%)	497 (71.8%)	0.039
Cyanoacrylate glue (mL)	1.0 (0.5-1.5)	1.0 (0.5-1.5)	0.790
Lauromacrogol (mL)	20.0 (10.0-30.0)	20.0 (10.0-40.0)	0.027
WBC (×10^9^/L)	4.7 (3.0-7.2)	3.7 (2.5-6.1)	<0.001
NLR	3.6 (2.3-6.2)	3.2 (2.0-5.1)	0.005
NEU%	70.8 (61.6-78.3)	70.0 (57.6-77.9)	0.025
NEU (×10^9^/L)	3.1 (1.9-5.5)	2.5 (1.5-4.5)	<0.001
LYM%	19.6 (12.9-26.8)	21.5 (14.8-30.1)	0.002
LYM (×10^9^/L)	0.8 (0.6-1.2)	0.8 (0.5-1.2)	0.062
RBC (×10^12^/L)	2.8 ± 0.8	3.1 ± 0.8	<0.001
HGB (g/L)	85.3 ± 25.9	93.0 ± 28.4	<0.001
PLT (×109/L)	73.4 (51.8-105.5)	60.2 (43.2-85.0)	<0.001
ALT (U/L)	23.1 (15.9-34.7)	27.0 (18.9-41.5)	<0.001
AST (U/L)	35.0 (23.1-55.4)	34.1 (24.9-48.4)	0.495
TBIL (*μ*mol/L)	24.3 (14.2-40.3)	19.8 (13.9-30.5)	0.001
DBIL (*μ*mol/L)	11.1 (6.2-20.9)	8.0 (5.5-13.1)	<0.001
ALB (g/L)	30.6 ± 6.2	31.9 ± 6.0	0.002
GGT (U/L)	57.3 (24.4-117.4)	27.6 (16.1-43.6)	<0.001
CHE (U/L)	3072 (2190-4063)	3091 (2320-4141)	0.237
TC (mm/L)	2.8 (2.1-3.5)	2.6 (2.2-3.4)	0.060
TG (mm/L)	0.7 (0.5-1.1)	0.6 (0.4-0.8)	<0.001
HDL-C (mm/L)	0.8 (0.5-1.0)	0.8 (0.6-1.1)	0.091
LDL-C (mm/L)	1.5 (1.1-2.0)	1.4 (1.1-1.8)	0.070
Cr (*μ*mol/L)	66.0 (57.0-79.0)	63.6 (53.4-73.7)	0.001
PT (s)	14.5 (13.3-16.4)	15.2 (14.0-16.7)	<0.001
INR	1.2 (1.1-1.4)	1.3 (1.2-1.4)	0.044
AFP (ng/mL)	2.7 (1.8-4.3)	4.3 (2.4-8.1)	<0.001
CTP class (A/B/C)	86/176/56	182/354/156	0.192
CTP score	8 (6-9)	8 (6-9)	0.092
MELD score	4 (0-7)	6 (4-9)	<0.001

WBC: white blood cell; NLR: neutrophil lymphocyte ratio; NEU%: neutrophilic granulocyte percentage; NEU: neutrophilic granulocyte; LYM%: lymphocyte percentage; LYM: lymphocytes; RBC: red blood cell; HGB: hemoglobin; PLT: platelet count; ALT: alanine transaminase; AST: aspartate aminotransferase; TBIL: total bilirubin; DBIL: direct bilirubin; ALB: albumin; GGT: gamma-glutamyl transpeptidase; CHE: cholinesterase; TC: total cholesterol; TG: total triglyceride; HDL-C: high-density lipoprotein cholesterol; LDL-C: low-density lipoprotein cholesterol; Cr: creatinine; PT: prothrombin time; INR: international normalized ratio; AFP: alpha fetoprotein.

**Table 2:** Factors associated with 5-year mortality.

Variable	Univariate analysis HR (95% CI)	*p*	Multivariate analysis HR (95% CI)	*p*
Age (year)	1.045 (1.034-1.056)	<0.001	1.048 (1.036-1.059)	<0.001
Sex	1.334 (1.043-1.706)	0.022		
WBC (×10^9^/L)	1.044 (1.013-1.077)	0.005		
NEU%	1.022 (1.012-1.032)	<0.001		
LYM%	0.968 (0.956-0.980)	<0.001	0.986 (0.973-0.999)	0.032
HGB (g/L)	0.989 (0.984-0.993)	<0.001	0.992 (0.987-0.997)	0.002
PLT (×109/L)	1.001 (0.999-1.004)	0.269		
ALT (U/L)	1.001 (0.999-1.002)	0.383		
AST (U/L)	1.000 (1.000-1.001)	0.260		
GGT (U/L)	1.001 (1.000-1.002)	0.027	1.002 (1.001-1.003)	<0.001
TBIL (*μ*mol/L)	1.005 (1.003-1.006)	<0.001		
DBIL (*μ*mol/L)	1.006 (1.004-1.009)	<0.001	1.003 (1.000-1.006)	0.025
ALB (g/L)	0.931 (0.914-0.950)	<0.001	0.964 (0.943-0.987)	0.002
Cr (*μ*mol/L)	1.003 (1.002-1.004)	<0.001		
INR	3.049 (2.100-4.428)	<0.001		
MELD score	1.073 (1.050-1.096)	<0.001	1.057 (1.032-1.083)	<0.001
Child-Pugh score	1.214 (1.150-1.281)	<0.001		

WBC: white blood cell; NEU%: neutrophilic granulocyte percentage; LYM%: lymphocytes percentage; HGB: hemoglobin; PLT: platelet count; ALT: alanine transaminase; AST: aspartate aminotransferase; GGT: gamma-glutamyl transpeptidase; TBIL: total bilirubin; DBIL: direct bilirubin; ALB: albumin; Cr: creatinine; INR: international normalized ratio.

## Data Availability

The data used to support the findings of this study are restricted by the Ethics Committee of Beijing Ditan Hospital, Capital Medical University, in order to protect patient privacy. Data are available from Pro. Hong-shan Wei (drwei@ccmu.edu.cn) for researchers who meet the criteria for access to confidential data.

## Abbreviations

HBV: Hepatitis B virus
HCV: Hepatitis C virus
AVB: Acute variceal bleeding
GI: Gastrointestinal
EIS: Endoscopic injection sclerotherapy
EVL: Endoscopic variceal ligation
WBC: White blood cell
RBC: Red blood cell
PLT: Platelet
LYM: Lymphocyte
NEU%: Neutrophilic granulocyte percentage
HGB: Hemoglobin
ALT: Alanine aminotransferase
AST: Aspartate transferase
GGT: Gamma-glutamyl transpeptidase
CHE: Cholinesterase
TBIL: Total bilirubin
ALB: Albumin
Cr: Creatinine
PT: Prothrombin time
AFP: Alpha-fetoprotein
GOV: Gastroesophageal varices
NSBBs: Nonselective beta blockers
HCC: Hepatocellular carcinoma.
